# Editorial: Natural Product Based Drugs that Control Obesity and Other Disorders that Trigger and Provoke Inflammation

**DOI:** 10.3389/fphar.2022.891496

**Published:** 2022-04-12

**Authors:** Fadia S. Youssef, Elisa Ovidi, Mahendra Rai

**Affiliations:** ^1^ Department of Pharmacognosy, Faculty of Pharmacy, Ain-Shams University, Cairo, Egypt; ^2^ Department for the Innovation in Biological, Agrofood and Forestal Systems, Tuscia University, Viterbo, Italy; ^3^ Department of Biotechnology, Sant Gadge Baba Amravati University, Amravati, India

**Keywords:** natural product, obesity, inflammation, secondary metabolites, ulcer

Obesity is a popular metabolic disorder triggered by excessive caloric consumption with a concomitant reduction in the expenditure of energy where it constitutes a gross health challenge threatening people all over the globe (Tallei et al., 2020). Unfortunately, it is closely related to the occurrence of various hazardous ailments such as chronic inflammation with consequent high levels of morbidity and mortality worldwide (Hoyt et al., 2014). Inflammation is a complex pathophysiological expression triggered by multiple molecular signals such as leukocytes, macrophages as well as mast cells. Chronic inflammation is characterized by being a prolonged inflammation that remains for multiple weeks to years without being treated causing a negative impact on tissue remodeling causing many disorders (Thabet et al., 2018). Thus obesity associated with chronic inflammation could be the cause of cardiovascular complications, diabetes, and cancer as well as neurodegenerative disorder (Ellulu et al., 2017). Natural products such as herbal drugs and marine organisms in addition to their secondary metabolites act as endless sources of promising drug leads that revealed significant anti-inflammatory as well as anti-obesity potential. They revealed higher safety margins, eco-friendly, and less expensive with respect to synthetic chemical entities, thus exploring additional drugs from natural origin is felt mandatory worldwide (Li et al., 2021). Thus, reviews and research articles gathered in this research topic shed the light on the role of natural products in the alleviation of obesity and inflammation and their related disorders.

In accordance with this concept, the study conducted by Shin et al. explained the effect of fermentation by lactic acid bacteria on ginsenosides identified in *Panax notoginseng* (PN), a traditional herbal medicine, and their efficacy to alleviate obesity a high-fat diet (HFD)-fed mouse model. Results illustrated that upon fermentation with *Lactobacillus plantarum*, the fermented PN extract displayed an altered ginsenoside profile with a considerable increase in lactate level that ultimately reflected in the obvious reduction in the food and calorie intake in contrast to unfermented extract via acting on multiple signaling pathways incorporated in appetite and energy consumption. Both the unfermented and fermented extracts modulated the gut microbial composition but they showed obvious differences in the relative abundances gut microbiota. The fermented extract showed a significant elevation in the relative abundance of *Akkermansia, Bacteroidetes, Coprococus, Dehalobacterium, Erysipelotrichaceae* and *Parpabacteroides* meanwhile *Erysipelotrichi, Allobaculum* and *Erysipelotrichale* were considerably low. Besides, the relative abundance of *Lactococcus* was higher in the fermented extract while the unfermented extract displayed higher level of *Bacteroides* relative to HFD group that ultimately reflected that the differences in the ginsenosides profile in *Panax notoginseng* as well as the gut microbial composition greatly contributed to the anti-obesity properties of the fermented extract.

In addition, the role of *Mytilus edulis* hydrolysate in the amelioration of lipopolysaccharide-galactosamine acute liver injury (LPS/D-GalN) and its consequent systemic inflammatory responses was discussed by Starikova et al. *Mytilus edulis* hydrolysate effectively protected mice versus LPS/D-GalN-triggered acute liver injury resulting in 100% survival rate upon constant administration of small doses of the drug in a subcutaneous pattern. The drug efficiently reduced Vascular Cell Adhesion Molecule-1 (VCAM-1), Interleukin-6 (IL-6) production in activated Human Umbilical Vein Endothelial Cells (HUVECs) *in vitro* with concomitant elevation in Nitric Oxide (NO) formation by HUVECs. However, it reduced Nitric Oxide Synthase (iNOS) expression as well as NO generation in murine peritoneal lavage cells. Furthermore, the ability of the extract to improve the endothelium barrier function and to decrease vascular permeability was evidenced *in vitro via* Electrical Cell-substrate impedance Sensor (ECIS) and *in vivo* using Miles assay which further consolidated the concept of adopting *Mytilus edulis* hydrolysate in case of endothelial dysfunction and uncontrolled inflammation.

In another interesting paper, Li et al. deliberated the role of glycyrrhizin, commonly known metabolites from Liquorice, as a promising drug in the alleviation of colon cancer cachexia. Cancer cachexia is considered as multifactorial symptom characterized by weight loss in addition to muscle wasting, resulted by many causes comprising decreased food intake, metabolic variation featured by, excessive catabolism and accompanied by inflammation (Biswas and Acharyya, 2020). Exosomes containing (Extracellular high mobility group box protein B1 (HMGB1) which is a crucial mediator incorporated in many acute and chronic inflammation pathogenesis led to muscle atrophy with a considerable reduction in myotube diameter and accompanied by an elevation in the expression of muscle atrophy-related proteins Atrogin1 and MuRF1 that play a main part in the occurrence and development of muscle wasting. HMGB1 stimulated the muscle atrophy mainly through TLR4/NF-κB pathway. Meanwhile, administration of the HMGB1 inhibitor glycyrrhizin could alleviates muscle wasting *in vitro* and attenuate the occurrence of cachexia *in vivo.*

Furthermore, the effectiveness of *Bryophyllum pinnatum* (Crassulaceae) leaf extract in the amelioration of acetic acid–induced chronic gastric ulcer and the gastroprotective activity of its major flavonoid, quercetin 3-O-α-L-arabinopyranosyl-(1→2)-O-α-L-rhamnopyranoside, Bp1 *versus* gastric lesions caused by indomethacin and ethanol and *in vivo* was highlighted by De Araújo et al. The extract effectively enhances the curing of gastric mucosa with considerable decrease in the ulceration index accompanied by enhancement of the antioxidant potential manifested by a pronounced elevation glutathione (GSH) levels and significant reduction in the levels of superoxide dismutase malondialdehyde (MDA). Concerning the anti-inflammatory markers, the extract significantly reduced the levels of tumor necrosis factor-α, interleukin-1β, and myeloperoxidase (MPO) whereas the levels of interleukin 10 were increased in the extract treated animals that is further evidenced by the cytoprotective effect detected by the histological analyzes. Significant amelioration was observed in the antioxidant and anti-inflammatory markers as well as the gastric lesions triggered by caused by indomethacin and ethanol upon treatment of the animals with 5 mg/kg of Bp1. However, the administration of the total extract displayed better results that undoubtedly relied upon the synergistic effect of all metabolites prevailing in the extract.

Additionally, a systematic review conducted by Palla et al. evaluated the effectiveness of many polyherbal combinations to reduce the severity of metabolic syndrome. After clearing all the collected data obtained by electronic search from any duplications, a total of 41 studies were found 24 of which were conducted in animal models meanwhile 15 were performed as clinical trials related to metabolic syndrome. SPICE (S = setting; P = population; I = intervention/what; C = comparison/controls E = evaluation/with what result) and SPIDER (S = Sample; P = phenomenon of interest/intervention; I = intervention size, D = design, E = evaluation/outcome R = research type; qualitative, quantitative or mixed type) models were used to evaluate clinical trials. The potential polyherbal combinations together with validation of the animal studies via systematic qualitative and quantitative analyses was stressed and provided a future direction to implement further research to prohibit and management of metabolic syndrome by polyherbal combinations.

In conclusion, this research topic consolidates the fact that the natural products can act as an everlasting mine offering new drug entities serving as drug leads for pharmaceutical industries combating obesity and chronic inflammation that are highly welcomed by many people all over the globe.
